# Antimicrobial resistance profiles of bacterial isolates from clinical specimens referred to Ethiopian Public Health Institute: analysis of 5-year data

**DOI:** 10.1186/s12879-023-08803-x

**Published:** 2023-11-15

**Authors:** Belay Tafa Regassa, Wagi Tosisa, Daniel Eshetu, Degefu Beyene, Abera Abdeta, Abebe Aseffa Negeri, Dejenie Shiferaw Teklu, Geremew Tasew, Begna Tulu, Tadesse Awoke

**Affiliations:** 1 Department of Medical Laboratory Sciences, College of Medicine and Health Sciences, Ambo University, Ambo, Ethiopia; 2 Yirgalem Hospital Medical College, Yirgalem, Sidama Regional State Ethiopia; 3 Ethiopian Public Health Institute, Addis Ababa, Ethiopia; 4 Department of Medical Laboratory Sciences, College of Medicine and Health Sciences, Bahir Dar University, Bahir Dar, Ethiopia; 5 Department of Epidemiology and Biostatistics, Institute of Public Health, College of Medicine and Health Sciences, University of Gondar, Gondar, Ethiopia

**Keywords:** Antibiotics, Antimicrobial resistance profile, Bacterial isolates, Clinical specimens, Multidrug resistance, MDR

## Abstract

**Background:**

Antimicrobial resistance is one of the common global public health problems. The emergence of antimicrobial resistance is multifactorial, and tackling its development is challenging. Consequently, infections caused by resistant bacteria are unresponsive to conventional drugs, resulting in prolonged and severe illnesses, higher mortality rates, and considerable healthcare costs. Therefore, understanding the antimicrobial resistance profiles of bacterial pathogens is essential to optimize treatments and reduce the risks associated with infections. This study aimed to determine the antimicrobial resistance patterns of bacterial isolates from different clinical specimens at the Ethiopian Public Health Institute (EPHI).

**Materials and methods:**

The retrospective cross-sectional study was conducted on the bacterial culture and antibiotic susceptibility reports of different clinical specimens referred to the Bacteriology Laboratory of EPHI from September 2015 to August 2019. Standard bacteriological techniques were used for the isolation and identification of the bacteria. Data were extracted from 840 patients’ records, which included the type of clinical sample cultured, the name of the bacteria, the representations of the antibiotics used for susceptibility testing, and the susceptibility results. Descriptive statistics were used to describe the bacterial isolates and the antimicrobial resistance profiles.

**Results:**

Eight types of clinical specimens were analyzed for bacterial isolates and urine specimens were the most analyzed. Ten different genera of bacteria were identified by culture. Almost all the isolates were gram-negative bacteria, while only one species of gram-positive (*Staphylococcus aureus*) was reported. Antibiotic sensitivity patterns were tested on 840 culture isolates. *Escherichia coli* strains revealed more than 57% resistance to seventeen antibiotics. *Klebsiella pneumoniae* showed nearly 70% or greater resistance rates for 17 of the antibiotics used. The overall detected multidrug resistance (MDR) was 64.29%. The highest MDR was reported in *Acinetobacter* strains (84%) followed by *K. pneumoniae* (80%).

**Conclusions:**

The multidrug resistance rates found in this study were alarming. Strengthening antimicrobial resistance surveillance at the national level is mandatory, and antimicrobial sensitivity testing should be accessible at local diagnostic centers.

**Supplementary Information:**

The online version contains supplementary material available at 10.1186/s12879-023-08803-x.

## Introduction

Antimicrobial resistance (AMR) is a phenomenon that occurs when different groups of microorganisms shift over a period and no longer respond to medicines that have been effective so far [1]. Although AMR is a natural process, the public health emergency due to the uncontrolled spread of this phenomenon is the consequence of overuse and/or misuse of antibiotics [2]. However, other driving factors are also mainly responsible for the increase in its prevalence. These include poor community hygiene, poor infection control in hospitals and clinics, the accumulation of antibiotics in the environment, and their use in the animal and food industries [3, 4]. Consequently, infections caused by resistant microorganisms often fail to respond to conventional treatment, resulting in prolonged illness, severe illness, the risk of disease spreading, a greater risk of death, and higher costs. In addition, new resistance mechanisms have emerged, making the latest generation of antibiotics virtually ineffective [5].

AMR is a global health and development threat that emerged as one of the major public health problems of the 21^st^ century and warns against the effective prevention and treatment of an ever-increasing range of infections [6]. The World Health Organization (WHO) has declared that AMR is one of humanity's top 10 global public health threats [1]. It has been noted over many decades that bacteria causing common or severe infections have developed resistance to every new antibiotic entering the market [3, 6]. Tackled by this reality, the need to prevent a rising global crisis in health care is crucial, requiring urgent multisectoral action to achieve the Sustainable Development Goals.

The World Health Organisation (WHO) has recognized for a long time that a better, more coordinated global effort is required to combat AMR. In 2001, the WHO Global Strategy for Containment of Antimicrobial Resistance provided an agenda of interferences to slow the emergence and reduce the distribution of antimicrobial-resistant microorganisms [7]. In 2012, WHO published ‘The Evolving Threat of Antimicrobial Resistance: Options for Action’ [8], proposing a combination of interventions that include strengthening health systems and surveillance, improving the use of antimicrobials in hospitals and the community, infection prevention and control, encouraging the development of appropriate new drugs and vaccines, and political commitment.

In 2014, following the proposed role of the surveillance system, WHO published the first global report on the surveillance of AMR, collecting experiences from national and international surveillance networks [9]. This report showed that surveillance data, where available, can be beneficial for orienting treatment choices, understanding AMR trends, identifying priority areas for interventions, and monitoring the impact of interventions to contain resistance. However, the lack of adequate surveillance in many parts of the world leaves significant gaps in existing knowledge of the distribution and extent of this phenomenon. In 2017, the WHO first published a list of 12 families of bacteria that pose the greatest threat to human health [10]. The WHO's list categorizes bacteria into three priority categories: critical, high, and medium priority, according to the need to develop new antibiotics to combat these pathogens [11].

The problem of antimicrobial resistance is not only the cause of the development of the resistance but also the transmission of the resistant strains from one person to another, especially in a health facility setting. The problem worsens in countries where poor sanitation makes transmitting the bacteria easy [12]. In addition, Ethiopia has a shortage of clinical microbiology laboratories to identify the specific etiologic agents and their antimicrobial susceptibility testing [13]. Therefore, treating most bacterial infections is usually done empirically, leading to an AMR emergency. AMR development in Ethiopia was mostly influenced locally by self-antibiotic prescription, lack of access to local antibiogram data, and low prescriber understanding of AMR [12, 13].

Understanding and acting on the local or national AMR situation is critical to gaining consensus on implementing appropriate interventions [14]. Furthermore, identifying the most common bacterial pathogens and their respective AMR profiles would be valuable to optimize treatment and reduce morbidity and mortality associated with infectious disease. Thus, up-to-date information on microbial resistance is needed at local and national levels to guide the rational use of the existing antimicrobials. Therefore, this study aimed to determine the antimicrobial resistance patterns of the bacterial isolate from different clinical specimens referred to EPHI.

## Materials and methods

### Study design, period, and setting

A retrospective cross-sectional study was conducted on bacterial culture and antibiotic susceptibility reports of different clinical specimens referred to the Bacteriology laboratory of the Ethiopian Public Health Institute (EPHI) between September 2015 and August 2019. The EPHI was established by the Council of Ministers regulation No. 301/2013. It recognizes the institute as an autonomous federal government office with its personality and is accountable to the Ethiopian Federal Minister of Health. The EPHI works with the mission of improving the health of the general public of the country through undertaking research on the priority health and nutrition issues for evidence-based information using and applying technology transfer, effective public health emergency management, establishing quality laboratory system, and training public health researchers and practitioners for the best public health interventions (https://ephi.gov.et/about-us/). Among the institute's vast activities of clinical laboratory services, strengthening national systems for referral specimen transportation, result delivery, and backup testing services are the ones (https://ephi.gov.et/laboratory-services/).

### Data extraction

A total of 840 records with complete information were selected for data extraction. The information extracted from the laboratory included the sex and age of patients, the type of clinical sample cultured, the name of bacteria isolated, names of antibiotics used for susceptibility testing, and the susceptibility results recorded in the laboratory report.

### Specimen collection, processing, and bacterial identifications

The standard operating procedures (SOPs) of collecting, storing, and transporting different clinical specimens were implemented, and specimens were referred to EPHI Bacteriology Laboratory. The collected clinical samples were delivered to the laboratory and processed following standard procedures. Bacteriological analyses were performed in the laboratory following SOPs adopted from the Clinical and Laboratory Standards Institute (CLSI) guidelines. Clinical samples, including urine, blood, sputum, wound/pus, cerebrospinal fluid, body fluid, discharge (ear/eye), and tracheal aspirate, were cultured. Each clinical sample employed standard microbiological culturing techniques. Specimens were inoculated into the appropriate isolation culture media and incubated at 35 to 37 °C conferring to standard protocols for each sample [15]. Bacterial identification was made mainly based on the colony characteristics, gram-stain reaction, and proper biochemical tests as per suitability according to CLSI guidelines and developed SOPs.

### Antimicrobial susceptibility testing

Antimicrobial sensitivity testing (AST) was performed using the Kirby Bauer disk diffusion technique on Mueller-Hinton agar. Standard discs with specified concentrations were used to detect the resistance patterns of each isolate. The plates were incubated overnight. After incubation was completed, the zone inhibition diameter was measured in millimeters (mm). The zones were interpreted as susceptible, intermediate, or resistant according to CLSI [16]. The definition of CDC was used in this study for multidrug resistance (MDR): resistance of bacterial isolates to at least one antibiotic in three or more drug classes (https://www.cdc.gov/narms/resources/glossary.html). The following standard antibiotics, with abbreviated names and disk contents in brackets, were used to test the resistance profiles of bacterial isolates: Amikacin (AMK) (30µg), Amoxicillin (AMX) (10µg), Amoxicillin/clavulanic acid (AMC) (20/10µg), Ampicillin (AMP) (10µg), Cefepime (FEP) (30µg), Cefamandole (CFM) (30µg), Cefotaxime (CTX) (30µg), Cefoxitin (FOX) (30µg), Ceftazidime (CAZ) (30µg), Ceftriaxone (CRO) (30µg), Cefuroxime (CXM) (30µg), Ciprofloxacin (CIP) (5µg), Chloramphenicol (CHL) (30µg), Clarithromycin (CLI) (15µg), Doripenem (DOR) (30µg), Erythromycin (ERY) (15µg), Gentamicin (GEN) (10µg), Meropenem (MEM) (10µg), Nalidixic acid (NAL) (30µg), Nitazoxanide (NIT) (300µg), Norfloxacin (NOR) 10µg), Oxacillin (OXA) (30µg), Penicillin (PEN) (10 units), Piperacillin (PIP) (100µg), Tazobactam (TZP) (100/10µg), Tobramycin (TOB) (10µg), Sulfamethoxazole-Trimethoprim (SXT) (1.25/23.75µg), Imipenem (IMP) (10µg), Cefazolin (KZ) (15µg), Tetracycline (TET) (15µg), Cefoperazone (CFP) (75µg) and Cefuroxime (CRX) (30µg).

### Quality control

American Typing Culture Collection isolates of *Escherichia coli* (ATCC-25922), *Staphylococcus aureus* (ATCC-25923), and *Pseudomonas aeruginosa* (ATCC-27853) were used as reference strains in the laboratory.

### Data analysis

The data entry was done using Microsoft Office Excel, and then it was imported to STATA version 14 for further analysis. Descriptive statistics were used to designate the demographic characteristics of the participants and the bacteriological and antimicrobial resistance profiles of the isolates.

## Results

### Profile of patients, clinical specimens, and bacterial isolates

Clinical specimens were collected from 840 patients; females accounted for 51.07% (*n* = 429). Most of the patients were in the age category of greater than 15 years old, 70.71% (*n* = 594). Eight different types of clinical specimens were analyzed for bacterial isolates, where urine specimens were the most analyzed, 64.52% (542/840). Analyzed by culture technique, ten different genera of bacteria were identified from the clinical samples. Almost all the isolates were gram-negative bacteria with only one species of gram-positive, *Staphylococcus aureus*. More than half of the gram-negative isolates reported were *Escherichia coli* (51.43%; 432/840). Please, refer to Table 1.

**Table 1:** Profile of patients, clinical specimens, and bacterial isolates

Profiles	Frequency	Percent
Sex		
Male	411	48.93
Female	429	51.07
Age category (in years)		
< 5	169	20.12
5 -15	77	9.17
> 15	594	70.71
Clinical samples		
Urine	542	64.52
Blood	160	19.05
Sputum	17	2.02
Wound/pus	72	8.57
Cerebrospinal fluid	5	0.60
Body fluid	16	1.90
Discharge (ear/eye)	9	1.07
Tracheal aspirate	19	2.26
Type of organism		
Gram-negative	826	98.33
Gram-positive	14	1.67
Bacterial isolates		
*Escherichia coli*	432	51.43
*Klebsiella pneumoniae*	225	26.79
*Klebsiella oxytoca*	16	1.90
*Klebsiella ozaenae*	17	2.02
*Enterobacter cloacae*	41	4.88
*Citrobacter diversus*	19	2.26
*Providencia alkalfacia*	2	0.24
*Providencia rettgeri*	3	0.36
*Proteus mirabilis*	8	0.95
*Proteus vulgaris*	1	0.12
*Providencia staurtti*	3	0.36
*Morganella morgani*	3	0.36
*Enterobacter aerogenes*^a^	2	0.24
*Citrobacter* spp	20	2.38
*Staphylococcus aureus*	14	1.67
*Enterobacter* spp	2	0.24
*Pseudomonas aeruginosa*	7	0.83
*Acinetobacter* spp	25	2.98

^a^Currently named *Klebsiella aerogenes*

### Bacterial isolates from various clinical specimens

Table 2 shows the type and frequency of bacterial isolates from the analyzed clinical specimens. *Escherichia coli* were identified from all types of clinical specimens with the majority from urine specimens, 83.3% (360/432). Similarily, *Klebsiella pneumoniae* was identified from all the analyzed specimens with the highest frequency from blood samples, 42.2% (95/225).

**Table 2:** Bacterial isolates from various clinical specimens

Bacterial isolates	Clinical specimens																
	**Urine**		**Blood**		**Sputum**		**Wound/pus**		**CSF**		**Body fluid**		**Discharge (ear/eye)**		**Tracheal aspirate**		**Total, N**
	**n**	**%**	**n**	**%**	**n**	**%**	**N**	**%**	**n**	**%**	**n**	**%**	**n**	**%**	**n**	**%**	
*Eschericia coli*	360	83.3	38	8.8	4	0.9	16	3.7	1	0.2	7	1.6	4	0.9	2	0.5	432
*Klebsiella pneumoniae*	92	40.9	95	42.2	7	3.1	18	8.0	3	1.3	5	2.2	2	0.9	3	1.3	225
*Klebsiella oxytoca*	7	43.8	6	37.5	0	0.0	2	12.5	0	0.0	1	6.3	0	0.0	0	0.0	16
*Klebsiella ozaenae*	10	58.8	4	23.5	0	0.0	3	17.6	0	0.0	0	0.0	0	0.0	0	0.0	17
*Enterobacter cloacae*	20	48.8	10	24.4	2	4.9	8	19.5	0	0.0	1	2.4	0	0.0	0	0.0	41
*Citrobacter diversus*	12	63.2	1	5.3	2	10.5	2	10.5	0	0.0	1	5.3	1	5.3	0	0.0	19
*Providencia alkalfacia*	0	0.0	0	0.0	0	0.0	1	50.0	0	0.0	0	0.0	1	50.0	0	0.0	2
*Providencia rettgeri*	2	66.7	1	33.3	0	0.0	0	0.0	0	0.0	0	0.0	0	0.0	0	0.0	3
*Proteus mirabilis*	3	37.5	0	0.0	0	0.0	4	50.0	0	0.0	0	0.0	1	12.5	0	0.0	8
*Proteus vulgaris*	1	100.0	0	0.0	0	0.0	0	0.0	0	0.0	0	0.0	0	0.0	0	0.0	1
*Providencia staurtti*	3	100.0	0	0.0	0	0.0	0	0.0	0	0.0	0	0.0	0	0.0	0	0.0	3
*Morganella morgani*	0	0.0	0	0.0	0	0.0	2	66.7	0	0.0	1	33.3	0	0.0	0	0.0	3
*Enterobacter aerogenes*	2	100.0	0	0.0	0	0.0	0	0.0	0	0.0	0	0.0	0	0.0	0	0.0	2
*Citrobacter* spp	15	75.0	1	5.0	2	10.0	2	10.0	0	0.0	0	0.0	0	0.0	0	0.0	20
*Staphylococcus aureus*	1	7.1	0	0.0	0	0.0	4	28.6	0	0.0	0	0.0	0	0.0	9	64.3	14
*Enterobacter* spp	2	100.0	0	0.0	0	0.0	0	0.0	0	0.0	0	0.0	0	0.0	0	0.0	2
*Pseudomonas aeruginosa*	0	0.0	0	0.0	0	0.0	3	42.9	0	0.0	0	0.0	0	0.0	4	57.1	7
*Acinetobacter* spp	12	48.0	4	16.0	0	0.0	7	28.0	1	4.0	0	0.0	0	0.0	1	4.0	25

### Antibiotic resistance patterns of bacterial isolates

The antibiotic resistance patterns were tested for 840 bacterial isolates over five years, from September 2015 to August 2019. As data are shown in Additional file 1: Table S1, different antibiotics were used to evaluate the resistance patterns of the bacterial isolates.

*Escherichia coli* strains were tested for 29 different antibiotics. For the fifteen antibiotics used, the *E. coli* strains revealed resistance of more than 57% (57.8% to 100%). The strains showed resistance of 98.9% and 100% to Ampicillin (AMP) and Cefuroxime (CXM), respectively. However, *E. coli* strains did not show resistance against Amoxicillin/clavulanic acid (AMC) and Chloramphenicol (CHL). The resistance patterns of *K. pneumoniae* were evaluated using 27 types of antibiotics. Nearly 70% and greater resistances were recorded for 17 antibiotics applied. On the other hand, lower resistances, less than 11%, were reported to a few antibiotics such as Amikacin (AMK), Doripenem (DOR), Erythromycin (ERY), and Imipenem (IMP) while no resistance was reported to Amoxicillin/clavulanic acid (AMC). *Acinetobacter* spp showed 100% resistance for the majority of the drugs: Cefepime (FEP), Cefotaxime (CTX), Ceftazidime (CAZ), Ceftriaxone (CRO), Cefuroxime (CXM), Nalidixic acid (NAL), Nitazoxanide (NIT), Tazobactam (TZP), and Tetracycline (TET). The resistance profiles of *S. aureus* strains were checked against eight drugs, and the strains showed no resistance for three antibiotics, such as Cefoxitin (FOX), Clarithromycin (CLI), and Gentamicin (GEN). Resistances of 70% and 50% were revealed against Penicillin (PEN) and Ciprofloxacin (CIP), respectively (Additional file 1: Table S1).

### Multidrug resistance (MDR) profiles of bacterial isolates

Overall, multidrug resistance (MDR) was 64.29% (540/840). The highest MDR was reported in *Acinetobacter* strains, 84% (21/25). The proportions of MDR - *K. pneumonia*e and MDR - *E. coli* were 80% (180/225) and 62.02% (268/432), respectively (Table 3).

**Table 3:** Multidrug resistance (MDR) profiles of bacterial isolates

Isolates	Total number of isolates	Resistance profiles to the number of classes of drugs				
		Resistance to none of the classes	Resistance to one class^a^	Resistance to two classes^a^	Resistance to three or more classes^a^	
					Number	Percent
*Eschericia coli*	432	24	57	83	268	62.04
*K.pneumoniae*	225	9	13	23	180	80.00
*K.oxytoca*	16	5	3	0	8	50.00
*K.ozaenae*	17	1	3	6	7	41.18
*Enterobacter cloacae*	41	6	7	9	19	46.34
*Citrobacter diversus*	19	3	2	2	12	63.16
*Providencia alkalfacia*	2	1	1	0	0	0.00
*Providencia rettgeri*	3	1	1	0	1	33.33
*Proteus mirabilis*	8	3	1	0	4	50.00
*Proteus vulgaris*	1	0	1	0	0	0.00
*Providencia staurtti*	3	1	0	0	2	66.67
*Morganella morgani*	3	0	0	1	2	66.67
*K.aerogenes*	2	1	0	0	1	50.00
*Citrobacter spp*	20	3	1	3	13	65.00
*S.aureus*	14	1	7	6	0	0.00
*Enterobacter spp*	2	0	0	1	1	50.00
*Pseudomonas aeruginosa*	7	0	4	2	1	14.29
*Acinetobacter spp*	25	0	0	4	21	84.00
Total	840	59	101	140	540	64.29

^a^Resistance of bacterial isolates to at least one antibiotic in a class of drugs

## Discussion

Antibiotic-resistant bacterial infections are among the most challenging public health concerns, especially in developing countries. The absence of effective antibiotic treatment will challenge clinicians to manage infectious diseases and their complications, particularly in immune-suppressed patients. This study assessed the antimicrobial resistance profiles of bacterial isolates from different clinical specimens referred to the Ethiopian Public Health Institute from the hospitals in Addis Ababa and the surrounding areas. This study showed that gram-negative bacteria were predominantly isolated from most clinical samples, which aligns with a study conducted in Northwest Ethiopia [13] and Tanzania [17]. The major bacterial isolate was recovered from the urine sample, which was 64.5% (542/840), followed by a blood sample of 19.05% (160/840). Therefore, most isolated bacteria in this review were gram-negative (98.33%), which varies from a study done in southwestern Ethiopia in which the most isolated bacteria (60%) were gram-positive [18]. The difference may be due to the large number of samples and types of clinical samples used in the studies used for this review. In addition, the isolated bacteria susceptibility test in the review was multidrug-resistant (85%), which is synonymous (81%) with the study done at a Referral Hospital in Northwest Ethiopia [19].

*E. coli* was the most commonly isolated bacterial pathogen, followed by *K. pneumoniae*; from all 840 isolates, *E. coli* accounts for 51.43%, regardless of specimen type this finding agrees with other studies done in India (53.3%) [20]. On the other hand, *E. coli* was the leading (51.43%) bacteria isolated among the others, not synonymous with a study done in Jimma University Specialized Hospital (JUSH) from April to August 2016 (25.4%), there is a high variation in percentage [21]. Therefore, the percentage difference may be due to sampling size variation. But more elevated than the study done in Hawassa tertiary hospital, Ethiopia (16.7%) [22], in Arbaminch General Hospital Ethiopia (40.5%) [23], Jimma, Ethiopia(42.0%) [24], Yemen(46.3%) [25]. The second most common gram-negative bacteria isolated from our study was *Klebsiella pneumoniae* (26.79%) which is higher than the study done in Saudi Arabia (20%) [26], Yemen (18.5%) [23], and India(17%) [27] but lower than the study done in Vinayaka Missions Medical College, India (41.55%) [28].

In our study, the overall prevalence MDR result showed a high rate of bacterial isolates, which was 64.29% (540/840). This result is lower than the studies done by Mengistu H. *et al. *[29], Ten Hove RJ. *et al*. [30], and Beyene D. *et al. *[31]. But in agreement with the studies done in Debre Markos Comprehensive Specialized Hospital (59%) [32], Gondar (64.6%) [32], and St. Paul’s Hospital Millennium Medical College (66.4%) [33], southern Ethiopia (69.9%) [34]. The most concerning part of this study was that a significant number of bacterial isolates showed drug resistance against the majority of antibiotics used for sensitivity testing. *E. coli*, *Klebsiella* spp, *Acinetobacte*r spp, and *Citrobacter* spp were highly resistant to commonly prescribed drugs like Sulfamethoxazole-Trimethoprim (Cotrimoxazole), Ceftriaxone, Ampicillin, Ciprofloxacin, Cefotaxime, Cefepime, and Ceftazidime. However, these bacteria are highly susceptible to Amoxicillin/clavulanic acid, Amikacin, Doripenem, Meropenem, and Imipenem. The overall observed high rate of MDR could be linked to irrational use and/or self-medication of antibiotics, possibly contributing to the resistance rates in the study area. This study has some limitations. Since our study was retrospective, it could not indicate the current antimicrobial resistance patterns of the isolates. This study also couldn’t determine whether the identified resistance was due to hospital-acquired or community-acquired.

## Conclusions

The most prevalent bacteria in this study, *Escherichia coli*, showed resistance of more than 57% to seventeen different antibiotics, while the next prevalent bacteria, *Klebsiella pneumoniae,* revealed nearly 70% or greater resistance rates. Overall, the multidrug resistance rates found in this study were alarming, 64.29%. Therefore, strengthening antimicrobial resistance surveillance at the national level, and antimicrobial sensitivity testing at local diagnostic centers are very important in reducing the challenges of antimicrobial resistance.

### Supplementary Information

**Additional file 1: Table S1. **Antibiotic resistance patterns of bacterial isolates.

## Data Availability

All the data are available with the corresponding author.
