# Correction: Antimicrobial resistance profiles of bacterial isolates from clinical specimens referred to ethiopian public health institute: analysis of 5-year data

**DOI:** 10.1186/s12879-023-08921-6

**Published:** 2024-01-02

**Authors:** Belay Tafa Regassa, Wagi Tosisa, Daniel Eshetu, Degefu Beyene, Abera Abdeta, Abebe Aseffa Negeri, Dejene Shiferaw Teklu, Geremew Tasew, Begna Tulu, Tadesse Awoke

**Affiliations:** 1 Department of Medical Laboratory Sciences, College of Medicine and Health Sciences, Ambo University, Ambo, Ethiopia; 2 Yirgalem Hospital Medical College, Yirgalem, Sidama Regional Ethiopia; 3 Ethiopian Public Health Institute, Addis Ababa, Ethiopia; 4 Department of Medical Laboratory Sciences, College of Medicine and Health Sciences, Bahir Dar University, Bahir Dar, Ethiopia; 5 Department of Epidemiology and Biostatistics, Institute of Public Health, College of Medicine and Health Sciences, University of Gondar, Gondar, Ethiopia

**Correction to: BMC Infectious Diseases (2023) 23:798**

10.1186/s12879-023-08803-x

The original publication of this article contains 2 incorrect author names. The incorrect and correct information is listed in this correction article. The original article has been updated.

**Incorrect**

Dejene Shiferew

Abebe Assefa

**Correct**

Dejenie Shiferaw Teklu

Abebe Aseffa Negeri

