# Supplementary material for: Antimicrobial resistance profiles of bacterial isolates from clinical specimens referred to Ethiopian Public Health Institute: analysis of 5-year data
Source: BMC Infect Dis. 2023 Nov 15;23:798. doi: 10.1186/s12879-023-08803-x (PMC10647041; doi:10.1186/s12879-023-08803-x)
Supplement: Supplementary file 1 — Additional file 1: Table S1. Antibiotic resistance patterns of bacterial isolates. [file 12879_2023_8803_MOESM1_ESM.docx]

**Table S1. Antibiotic resistance patterns of bacterial isolates**

| **Bacterial isolates** | **Resistance patterns to antibiotics** | | | | | | | | | | | | | | | | | | | | | | | | | | | | | | | |
| --- | --- | --- | --- | --- | --- | --- | --- | --- | --- | --- | --- | --- | --- | --- | --- | --- | --- | --- | --- | --- | --- | --- | --- | --- | --- | --- | --- | --- | --- | --- | --- | --- |
|  | **AMK** | | | | **AMX** | | | | **AMC** | | | | **AMP** | | | | **FEP** | | | | **CFM** | | | | **CTX** | | | | **FOX** | | | |
|  | **I** | **R** | **S** | **%R** | **I** | **R** | **S** | **%R** | **I** | **R** | **S** | **%R** | **I** | **R** | **S** | **%R** | **I** | **R** | **S** | **%R** | **I** | **R** | **S** | **%R** | **I** | **R** | **S** | **%R** | **I** | **R** | **S** | **%R** |
| *Eschericia coli* | 21 | 16 | 394 | 3.7 | 0 | 4 | 1 | 80 | 58 | 0 | 216 | 0 | 0 | 187 | 2 | 98.9 | 15 | 119 | 60 | 61.3 | 0 | 1 | 2 | 33.3 | 5 | 223 | 148 | 59.3 | 8 | 42 | 178 | 18.4 |
| *Klebsiella pneumoniae* | 18 | 23 | 178 | 10.5 | - | - | - | - | 38 | 0 | 141 | 0 | 0 | 30 | 1 | 96.8 | 4 | 91 | 17 | 81.3 | - | - | - | - | 2 | 154 | 24 | 85.6 | 0 | 24 | 78 | 23.5 |
| *Klebsiella oxytoca* | 0 | 2 | 14 | 12.5 | - | - | - | - | 1 | 0 | 8 | 0 | - | - | - | - | 0 | 1 | 0 | 100 | - | - | - | - | 0 | 8 | 7 | 53.3 | 0 | 2 | 13 | 13.3 |
| *Klebsiella ozaenae* | 2 | 0 | 14 | 0.0 | - | - | - | - | 1 | 0 | 10 | 0 | 0 | 1 | 0 | 100 | 0 | 2 | 0 | 100 | - | - | - | - | 1 | 8 | 6 | 53.3 | 0 | 2 | 11 | 15.4 |
| *Enterobacter cloacae* | 1 | 2 | 36 | 5.1 | - | - | - | - | 2 | 0 | 16 | 0 | 0 | 2 | 0 | 100 | 1 | 11 | 5 | 64.7 | - | - | - | - | 0 | 24 | 14 | 63.2 | 0 | 14 | 8 | 63.6 |
| *Citrobacter diversus* | 5 | 0 | 14 | 0.0 | - | - | - | - | 0 | 0 | 13 | 0 | - | - | - | - | - | - | - | - | - | - | - | - | 0 | 12 | 7 | 63.2 | 0 | 5 | 14 | 26.3 |
| *Providencia alkalfacia* | 0 | 0 | 2 | 0.0 | - | - | - | - | 0 | 0 | 1 | 0 | - | - | - | - | - | - | - | - | - | - | - | - | 0 | 0 | 2 | 0 | 1 | 0 | 1 | 0 |
| *Providencia rettgeri* | 0 | 0 | 3 | 0.0 | - | - | - | - | 0 | 0 | 1 | 0 | - | - | - | - | 0 | 0 | 1 | 0 | - | - | - | - | 0 | 1 | 1 | 50 | 0 | 1 | 1 | 50 |
| *Proteus mirabilis* | 0 | 1 | 6 | 14.3 | - | - | - | - | 0 | 0 | 2 | 0 | - | - | - | - | 0 | 2 | 0 | 100 | - | - | - | - | 0 | 2 | 4 | 33.3 | 0 | 0 | 5 | 0 |
| *Proteus vulgaris* | - | - | - | - | - | - | - | - | - | - | - | - | - | - | - | - | 0 | 0 | 1 | 0 | - | - | - | - | 0 | 0 | 1 | 0 | - | - | - | - |
| *Providencia staurtti* | 0 | 0 | 3 | 0.0 | - | - | - | - | 0 | 2 | 1 | 66.7 | - | - | - | - | - | - | - | - | - | - | - | - | 0 | 2 | 1 | 66.7 | 0 | 0 | 3 | 0 |
| *Morganella morgani* | 1 | 0 | 2 | 0.0 | - | - | - | - | 0 | 3 | 0 | 100 | 0 | 1 | 0 | 100 | 0 | 0 | 1 | 0 | - | - | - | - | 0 | 2 | 0 | 100 | 0 | 1 | 1 | 50 |
| *Enterobacter aerogenes* | 0 | 0 | 2 | 0.0 | - | - | - | - | 1 | 0 | 1 | 0 | - | - | - | - | - | - | - | - | - | - | - | - | 0 | 1 | 1 | 50 | 0 | 0 | 2 | 0 |
| *Citrobacter* spp | 0 | 0 | 20 | 0.0 | - | - | - | - | 0 | 6 | 5 | 54.5 | - | - | - | - | 1 | 9 | 0 | 90 | - | - | - | - | 0 | 13 | 6 | 68.4 | 2 | 5 | 3 | 50 |
| *Staphylococcus aureus* | - | - | - | - | - | - | - | - | - | - | - |  | - | - | - | - | - | - | - | - | - | - | - | - | - | - | - | - | 0 | 0 | 5 | 0 |
| *Enterobacter* spp | - | - | - | - | - | - | - | - | - | - | - | - | 0 | 2 | 0 | 100 | - | - | - | - | - | - | - | - | - | - | - | - | - | - | - | - |
| *Pseudomonas aeruginosa* | 1 | 0 | 5 | 0.0 | - | - | - | - | - | - | - | - | - | - | - | - | 2 | 1 | 4 | 14.3 | - | - | - | - | - | - | - | - | - | - | - | - |
| *Acinetobacter* spp | 0 | 7 | 12 | 36.8 | - | - | - | - | 1 | 0 | 0 | 0 | - | - | - | - | 0 | 17 | 0 | 100 | - | - | - | - | 0 | 9 | 0 | 100 | - | - | - | - |

**Table S1 Continued…**

| **Bacterial isolates** | **Resistance patterns to antibiotics** | | | | | | | | | | | | | | | | | | | | | | | | | | | | | | | |
| --- | --- | --- | --- | --- | --- | --- | --- | --- | --- | --- | --- | --- | --- | --- | --- | --- | --- | --- | --- | --- | --- | --- | --- | --- | --- | --- | --- | --- | --- | --- | --- | --- |
|  | **CAZ** | | | | **CRO** | | | | **CXM** | | | | **CHL** | | | | **CIP** | | | | **CLI** | | | | **DOR** | | | | **ERY** | | | |
|  | **I** | **R** | **S** | **%R** | **I** | **R** | **S** | **%R** | **I** | **R** | **S** | **%R** | **I** | **R** | **S** | **%R** | **I** | **R** | **S** | **%R** | **I** | **R** | **S** | **%R** | **I** | **R** | **S** | **%R** | **I** | **R** | **S** | **%R** |
| *Eschericia coli* | 10 | 247 | 170 | 57.8 | 2 | 129 | 59 | 67.9 | 0 | 2 | 0 | 100 | 0 | 0 | 3 | 0 | 17 | 283 | 127 | 66.3 | - | - | - | - | 0 | 1 | 138 | 0.7 | 1 | 1 | 139 | 0.7 |
| *Klebsiella pneumoniae* | 3 | 189 | 29 | 85.5 | 1 | 99 | 14 | 86.8 | 0 | 6 | 0 | 100 | 0 | 1 | 0 | 100 | 29 | 120 | 72 | 54.3 | - | - | - | - | 0 | 6 | 67 | 8.2 | 0 | 6 | 67 | 8.2 |
| *Klebsiella oxytoca* | 0 | 9 | 7 | 56.3 | - | - | - |  | - | - | - |  | - | - | - | - | 0 | 5 | 10 | 33.3 | - | - | - | - | - | - | - | - | - | - | - | - |
| *Klebsiella ozaenae* | 0 | 9 | 6 | 60.0 | 0 | 2 | 0 | 100 | 0 | 3 | 0 | 100 | - | - | - | - | 3 | 5 | 8 | 31.3 | - | - | - | - | - | - | - | - | - | - | - | - |
| *Enterobacter cloacae* | 0 | 22 | 16 | 57.9 | 1 | 14 | 4 | 73.7 | 0 | 1 | 0 | 100 | - | - | - | - | 7 | 11 | 22 | 27.5 | - | - | - | - | 0 | 0 | 16 | 0.0 | 0 | 0 | 16 | 0.0 |
| *Citrobacter diversus* | 0 | 12 | 7 | 63.2 | - | - | - | - | - | - | - | - | - | - | - | - | 3 | 9 | 7 | 47.4 | - | - | - | - | - | - | - | - | - | - | - | - |
| *Providencia alkalfacia* | 0 | 0 | 2 | 0.0 | - | - | - | - | - | - | - | - | - | - | - | - | 0 | 0 | 2 | 0.0 | - | - | - | - | - | - | - | - | - | - | - | - |
| *Providencia rettgeri* | 0 | 1 | 2 | 33.3 | - | - | - | - | - | - | - | - | - | - | - | - | 0 | 1 | 2 | 33.3 | - | - | - | - | - | - | - | - | - | - | - | - |
| *Proteus mirabilis* | 0 | 2 | 4 | 33.3 | 0 | 1 | 0 | 100 | 0 | 1 | 0 | 100 | - | - | - | - | 1 | 4 | 3 | 50.0 | - | - | - | - | - | - | - | - | - | - | - | - |
| *Proteus vulgaris* | 0 | 0 | 1 | 0.0 | 0 | 0 | 1 | 0.0 | 0 | 0 | 1 | 0 | - | - | - | - | - | - | - | - | - | - | - | - | - | - | - | - | - | - | - | - |
| *Providencia staurtti* | 0 | 2 | 1 | 66.7 | - | - | - |  | - | - | - | - | - | - | - | - | 0 | 1 | 2 | 33.3 | - | - | - | - | - | - | - | - | - | - | - | - |
| *Morganella morgani* | 1 | 1 | 0 | 50.0 | 0 | 1 | 0 | 100 | - | - | - | - | - | - | - | - | 0 | 2 | 1 | 66.7 | - | - | - | - | - | - | - | - | - | - | - | - |
| *Enterobacter aerogenes* | 0 | 1 | 1 | 50.0 | - | - | - |  | - | - | - | - | - | - | - | - | 0 | 0 | 2 | 0.0 | - | - | - | - | - | - | - | - | - | - | - | - |
| *Citrobacter* spp | 0 | 12 | 8 | 60.0 | 1 | 8 | 1 | 80 | - | - | - | - | - | - | - | - | 0 | 13 | 7 | 65.0 | - | - | - | - | 0 | 0 | 9 | 0.0 | 0 | 0 | 9 | 0.0 |
| *Staphylococcus aureus* | - | - | - |  | - | - | - |  | - | - | - | - | - | - | - | - | 1 | 3 | 2 | 50.0 | 3 | 0 | 9 | 0 | - | - | - | - | 3 | 2 | 7 | 16.7 |
| *Enterobacter* spp | - | - | - |  | - | - | - |  | - | - | - | - | - | - | - | - | 0 | 2 | 0 | 100 | - | - | - |  | - | - | - | - | - | - | - | - |
| *Pseudomonas aeruginosa* | 1 | 2 | 1 | 50.0 | - | - | - |  | - | - | - | - | - | - | - | - | 0 | 2 | 5 | 28.6 | - | - | - |  | - | - | - | - | - | - | - | - |
| *Acinetobacter* spp | 0 | 16 | 0 | 100 | 0 | 9 | 0 | 100 | 0 | 3 | 0 | 100 | - | - | - | - | 0 | 20 | 2 | 90.9 | - | - | - |  | - | - | - | - | - | - | - | - |

**Table S1 Continued…**

| **Bacterial isolates** | **Resistance patterns to antibiotics** | | | | | | | | | | | | | | | | | | | | | | | | | | | | | | | | | | | |
| --- | --- | --- | --- | --- | --- | --- | --- | --- | --- | --- | --- | --- | --- | --- | --- | --- | --- | --- | --- | --- | --- | --- | --- | --- | --- | --- | --- | --- | --- | --- | --- | --- | --- | --- | --- | --- |
|  | **GEN** | | | | **MEM** | | | | **NAL** | | | | **NIT** | | | | **NOR** | | | | **OXA** | | | | **PEN** | | | | **PIP** | | | | **TZP** | | | |
|  | I | R | S | %R | I | R | S | %R | I | R | S | %R | I | R | S | %R | I | R | S | %R | I | R | S | %R | I | R | S | %R | I | R | S | %R | I | R | S | %R |
| *Eschericia coli* | 8 | 120 | 295 | 28 | 4 | 9 | 417 | 2.1 | 0 | 1 | 0 | 100 | 3 | 3 | 22 | 11 | 1 | 225 | 124 | 64 | - | - | - | - | - | - | - | - | 7 | 107 | 34 | 72 | 12 | 32 | 147 | 16.8 |
| *Klebsiella pneumoniae* | 5 | 149 | 62 | 69 | 9 | 32 | 180 | 15 | 0 | 2 | 0 | 100 | 0 | 19 | 4 | 83 | 12 | 45 | 68 | 36 | - | - | - | - | - | - | - | - | 1 | 71 | 13 | 84 | 9 | 43 | 56 | 39.8 |
| *Klebsiella oxytoca* | 0 | 6 | 10 | 38 | 0 | 2 | 14 | 13 | - | - | - | - | - | - | - | - | 0 | 6 | 9 | 40 | - | - | - | - | - | - | - | - | - | - | - | - | 0 | 1 | 0 | 100 |
| *Klebsiella ozaenae* | 0 | 5 | 10 | 33 | 0 | 2 | 15 | 12 | - | - | - | - | 0 | 2 | 0 | 100 | 2 | 3 | 8 | 23 | - | - | - | - | - | - | - | - | 0 | 4 | 0 | 100 | - | - | - | - |
| *Enterobacter cloacae* | 0 | 17 | 22 | 44 | 0 | 1 | 40 | 2.4 | 0 | 0 | 1 | 0 | 1 | 0 | 0 | 0 | 0 | 7 | 23 | 23 | - | - | - | - | - | - | - | - | 0 | 13 | 4 | 77 | 1 | 1 | 14 | 6.3 |
| *Citrobacter diversus* | 2 | 8 | 9 | 42 | 1 | 0 | 18 | 0 | - | - | - | - | - | - | - | - | 1 | 9 | 9 | 47 | - | - | - | - | - | - | - | - | - | - | - | - | - | - | - | - |
| *Providencia alkalfacia* | 0 | 0 | 2 | 0 | 0 | 0 | 2 | 0 | - | - | - | - | - | - | - | - | 0 | 0 | 2 | 0 | - | - | - | - | - | - | - | - | - | - | - | - | - | - | - | - |
| *Providencia rettgeri* | 0 | 0 | 3 | 0 | 0 | 0 | 3 | 0 | - | - | - | - | - | - | - | - | 0 | 1 | 1 | 50 | - | - | - | - | - | - | - | - | 0 | 1 | 0 | 100 | - | - | - | - |
| *Proteus mirabilis* | 0 | 2 | 6 | 25 | 0 | 2 | 6 | 25 | - | - | - | - | 0 | 1 | 0 | 100 | 0 | 1 | 4 | 20 | - | - | - | - | - | - | - | - | 0 | 2 | 1 | 67 | - | - | - | - |
| *Proteus vulgaris* | - | - | - |  | - | - | - |  | - | - | - | - | - | - | - | - | - | - | - | - | - | - | - | - | - | - | - | - | - | - | - | - | - | - | - | - |
| *Providencia staurtti* | 0 | 1 | 2 | 33 | 0 | 0 | 3 | 0 | - | - | - | - | - | - | - | - | 0 | 1 | 2 | 33 | - | - | - | - | - | - | - | - | - | - | - | - | - | - | - | - |
| *Morganella morgani* | 0 | 2 | 1 | 67 | 0 | 1 | 2 | 33 | - | - | - | - | - | - | - | - | 0 | 1 | 1 | 50 | - | - | - | - | - | - | - | - | 0 | 1 | 0 | 100 | - | - | - | - |
| *Enterobacter aerogenes* | 0 | 1 | 1 | 50 | 0 | 0 | 2 | 0 | - | - | - | - | - | - | - | - | 0 | 0 | 2 | 0 | - | - | - | - | - | - | - | - | - | - | - | - | - | - | - | - |
| *Citrobacter* spp | 0 | 9 | 11 | 45 | 0 | 1 | 19 | 5 | - | - | - | - | 0 | 0 | 1 | 0 | 1 | 10 | 6 | 59 | - | - | - | - | - | - | - | - | 0 | 8 | 1 | 89 | 0 | 1 | 9 | 10 |
| *Staphylococcus aureus* | 1 | 0 | 8 | 0 | - | - | - | - | - | - | - | - | - | - | - | - | - | - | - | - | 0 | 2 | 8 | 20 | 0 | 10 | 3 | 77 | - | - | - | - | - | - | - | - |
| *Enterobacter* spp | - | - | - |  | - | - | - | - | - | - | - | - | 0 | 0 | 2 | 0 | - | - | - | - | - | - | - | - | - | - | - | - | - | - | - | - | - | - | - | - |
| *Pseudomonas aeruginosa* | 0 | 0 | 2 | 0 | 0 | 6 | 0 | 100 | - | - | - | - | - | - | - | - | - | - | - | - | - | - | - | - | - | - | - | - | - | - | - | - | - | - | - | - |
| *Acinetobacter* spp | 2 | 13 | 6 | 62 | 3 | 19 | 2 | 79 | 0 | 3 | 0 | 100 | 0 | 7 | 0 | 100 | - | - | - | - | - | - | - | - | - | - | - | - | - | - | - | - | 0 | 2 | 0 | 100 |

**Table S1 Continued…**

| **Bacterial isolates** | **Resistance patterns to antibiotics** | | | | | | | | | | | | | | | | | | | | | | | | | | | |
| --- | --- | --- | --- | --- | --- | --- | --- | --- | --- | --- | --- | --- | --- | --- | --- | --- | --- | --- | --- | --- | --- | --- | --- | --- | --- | --- | --- | --- |
|  | TOB | | | | SXT | | | | IMP | | | | KZ | | | | TET | | | | CFP | | | | CRX | | | |
|  | **I** | **R** | **S** | **%R** | **I** | **R** | **S** | **%R** | **I** | **R** | **S** | **%R** | **I** | **R** | **S** | **%R** | **I** | **R** | **S** | **%R** | **I** | **R** | **S** | **%R** | **I** | **R** | **S** | **%R** |
| *Eschericia coli* | 2 | 44 | 106 | 29 | 1 | 324 | 96 | 77 | 0 | 1 | 140 | 1 | 13 | 98 | 30 | 70 | 6 | 110 | 25 | 78 | 11 | 111 | 107 | 49 | 0 | 94 | 47 | 66.7 |
| *Klebsiella pneumoniae* | 4 | 58 | 25 | 67 | 1 | 193 | 24 | 89 | 0 | 6 | 67 | 8 | 2 | 65 | 6 | 89 | 5 | 55 | 18 | 71 | 3 | 90 | 16 | 83 | 0 | 61 | 12 | 83.6 |
| *Klebsiella oxytoca* | - | - | - | - | 0 | 10 | 6 | 63 | - | - | - | - | - | - | - | - | - | - | - | - | 0 | 7 | 8 | 47 | - | - | - | - |
| *Klebsiella ozaenae* | 0 | 2 | 1 | 67 | 0 | 12 | 5 | 71 | - | - | - | - | - | - | - | - | 0 | 3 | 0 | 100 | 0 | 8 | 7 | 53 | - | - | - | - |
| *Enterobacter cloacae* | 1 | 9 | 9 | 47 | 0 | 25 | 15 | 63 | 0 | 0 | 16 | 0 | 0 | 14 | 2 | 88 | 1 | 8 | 6 | 53 | 2 | 11 | 10 | 48 | 0 | 12 | 4 | 75 |
| *Citrobacter diversus* | - | - | - | - | 0 | 15 | 4 | 79 | - | - | - | - | - | - | - | - | - | - | - | - | 0 | 12 | 7 | 63 | - | - | - | - |
| *Providencia alkalfacia* | - | - | - | - | 0 | 0 | 2 | 0 | - | - | - | - | - | - | - | - | - | - | - | - | 0 | 0 | 2 | 0 | - | - | - | - |
| *Providencia rettgeri* | - | - | - | - | 0 | 1 | 1 | 50 | - | - | - | - | - | - | - | - | - | - | - | - | 0 | 1 | 1 | 50 | - | - | - | - |
| *Proteus mirabilis* | - | - | - | - | 1 | 5 | 2 | 63 | - | - | - | - | - | - | - | - | 0 | 1 | 0 | 100 | 0 | 3 | 4 | 43 | - | - | - | - |
| *Proteus vulgaris* | - | - | - | - | - | - | - | - | - | - | - | - | - | - | - | - | - | - | - | - | 0 | 1 | 0 | 100 | - | - | - | - |
| *Providencia staurtti* | - | - | - | - | 0 | 2 | 1 | 67 | - | - | - | - | - | - | - | - | - | - | - | - | 0 | 2 | 1 | 67 | - | - | - | - |
| *Morganella morgani* | 0 | 1 | 0 | 100 | 0 | 3 | 0 | 100 | - | - | - | - | - | - | - | - | - | - | - | - | 1 | 2 | 0 | 67 | - | - | - | - |
| *Enterobacter aerogenes* | - | - | - | - | 0 | 1 | 1 | 50 | - | - | - | - | - | - | - | - | - | - | - | - | 0 | 1 | 1 | 50 | - | - | - | - |
| *Citrobacter* spp | 0 | 6 | 3 | 67 | 0 | 14 | 6 | 70 | 0 | 0 | 9 | 0 | 0 | 7 | 1 | 88 | 0 | 7 | 1 | 88 | 0 | 5 | 5 | 50 | 0 | 8 | 1 | 88.9 |
| *Staphylococcus aureus* | - | - | - | - | 0 | 3 | 6 | 33 | - | - | - | - | - | - | - | - | - | - | - | - | - | - | - | - | - | - | - | - |
| *Enterobacter* spp | - | - | - | - | 0 | 1 | 1 | 50 | - | - | - | - | - | - | - | - | - | - | - | - | - | - | - | - | - | - | - | - |
| *Pseudomonas aeruginosa* | 0 | 2 | 4 | 33 | - | - | - | - | - | - | - | - | - | - | - | - | - | - | - | - | - | - | - | - | - | - | - | - |
| *Acinetobacter* spp | 2 | 7 | 7 | 44 | 2 | 16 | 2 | 80 | - | - | - | - | - | - | - | - | 0 | 4 | 0 | 100 | - | - | - | - | - | - | - | - |

Amikacin (AMK) (30µg); Amoxicillin (AMX) (10µg); Amoxicillin/clavulanic acid (AMC) (20/10µg); Ampicillin (AMP) (10µg); Cefepime (FEP) (30µg); Cefamandole (CFM) (30µg); Cefotaxime (CTX) (30µg); Cefoxitin (FOX) (30µg); Ceftazidime (CAZ) (30µg); Ceftriaxone (CRO) (30µg); Cefuroxime (CXM) (30µg); Ciprofloxacin (CIP) (5µg); Chloramphenicol (CHL) (30µg); Clarithromycin (CLI) (15µg); Doripenem (DOR) (30µg); Erythromycin (ERY) (15µg); Gentamicin (GEN) (10µg); Meropenem (MEM) (10µg); Nalidixic acid (NAL) (30µg); Nitazoxanide (NIT) (300µg); Norfloxacin (NOR) 10µg); Oxacillin (OXA) (30µg); Penicillin (PEN) (10 units); Piperacillin (PIP) (100µg); Tazobactam (TZP) (100/10µg); Tobramycin (TOB) (10µg); Sulfamethoxazole-Trimethoprim (SXT) (1.25/23.75µg); Imipenem (IMP) (10µg); Cefazolin (KZ) (15µg); Tetracycline (TET) (15µg); Cefoperazone (CFP) (75µg); Cefuroxime (CRX) (30µg); I = Intermediate; R = Resistant; S = Susceptible.
